# Oblique Osteotomy as an Alternative for the Management of Femoral Shaft Non-union

**DOI:** 10.7759/cureus.79915

**Published:** 2025-03-02

**Authors:** Thylane E Vancastell, Matija Krkovic

**Affiliations:** 1 Trauma and Orthopaedic Surgery, Addenbrooke's Hospital, Cambridge University Hospitals NHS Foundation Trust, Cambridge, GBR

**Keywords:** complications in femoral shaft fractures, deep wound infection, delayed fracture union, femoral non-union, femur shaft fractures, intramedullary femoral nail, limb reconstruction, oblique osteotomy, poller screws, revision surgery femoral shaft fracture

## Abstract

Femoral non-unions, especially those with transverse non-union lines, pose considerable challenges in orthopaedic practice and often require multiple revision surgeries to achieve union. We describe the management of an infected non-union of the proximal femoral shaft in a patient who initially underwent fixation with an antegrade nail. The removal of the nail led to an additional iatrogenic fracture. The non-union and the fracture were stabilised with a plate and cerclage wires. The plate fixation failed seven weeks later. The patient, a 50-year-old female, had multiple comorbidities and was undergoing immunosuppressive treatment. To facilitate interfragmentary compression, the non-union was resected by creating oblique osteotomy lines, and biplanar poller screws were applied in both fragments around a retrograde femoral nail. The revision fixation was complicated by an acute infection, which required modified irrigation negative pressure wound therapy and an appropriate course of antibiotics for 12 weeks. The patient commenced weight bearing as tolerated three days post-surgery, and the non-union has united in the X-rays in the follow-up appointments between seven to 11 months after the revision fixation.

## Introduction

Femoral fractures usually heal uneventfully. However, non-union remains the most common post-fracture fixation complication, with an incidence of 5% to 10% [1,2]. Non-union procedures are challenging [3] and have a high failure rate [4]. In our experience, transverse fractures of long bones present a higher risk of non-union than other fracture types. This increased risk may be attributed to the limited surface area for bone contact, which can impede the healing process. While specific studies focusing solely on transverse fractures are limited, general principles of fracture healing suggest that fractures with smaller contact surfaces are more prone to non-union [5]. For instance, fractures with significant bone loss or gaps greater than 3 mm have been identified as contributing factors to non-union [5]. 

Oblique osteotomy with the use of biplanar poller screws offers mechanical and biological advantages by increasing bone surface area for healing and generating interfragmentary compression [6]. In our opinion, achieving the same in a horizontal osteotomy is almost impossible due to the very high precision required in the insertion of the poller screws.

## Case presentation

**Figure 1:**
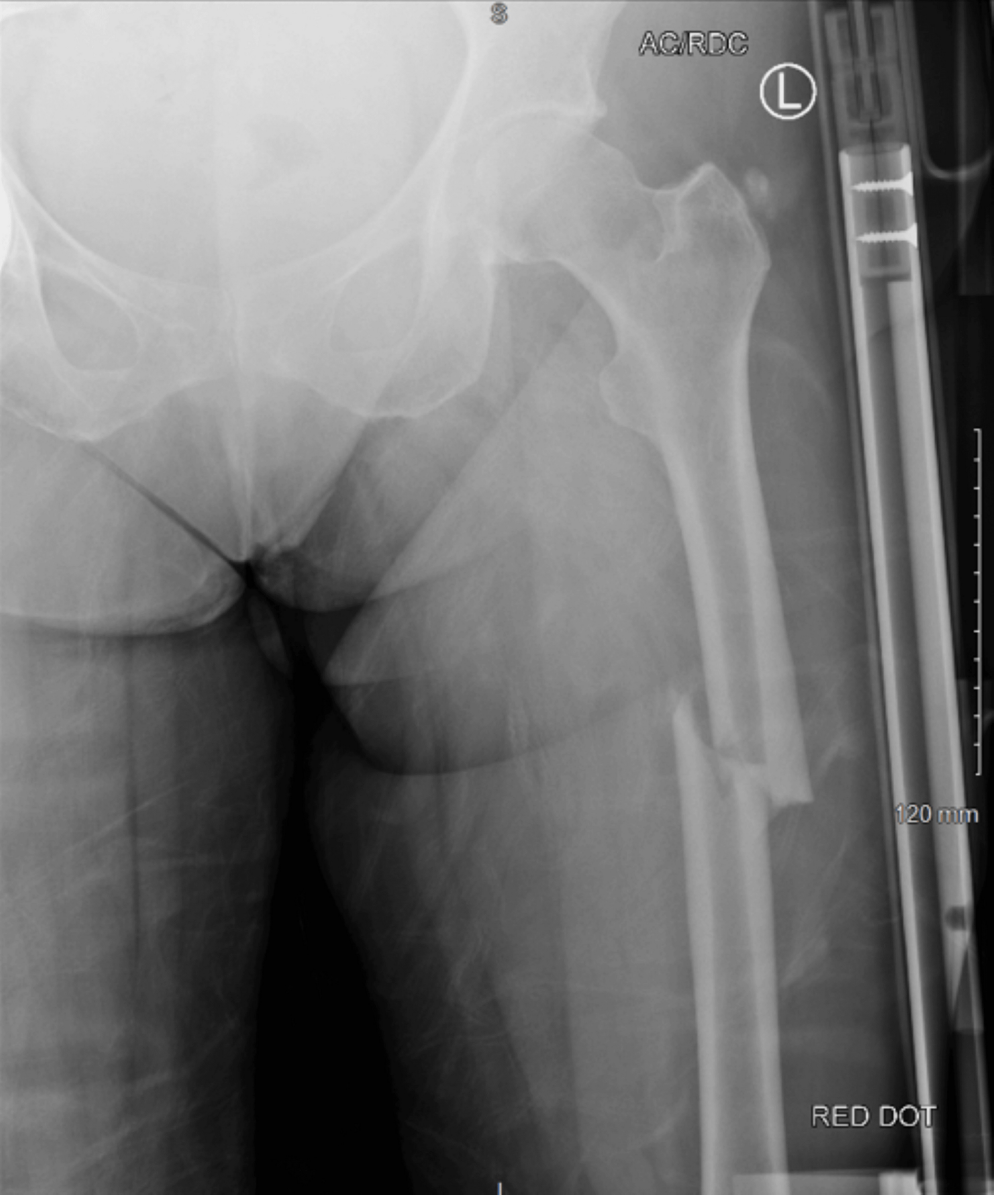
Anteroposterior X-ray of the left femur after the accident from the hospital providing primary care

A 50-year-old female patient fell in the kitchen and sustained a closed left proximal femoral fracture (Figure 1).

**Figure 2:**
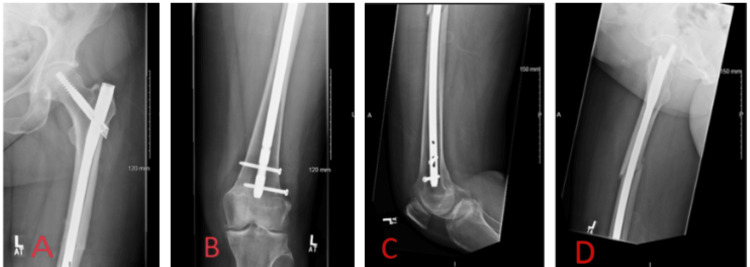
Postoperative X-rays after the first surgery from the hospital providing primary care A: Anteroposterior X-ray of the left proximal femur; B: Anteroposterior X-ray of the left distal femur; C: Lateral X-ray of the left distal femur; D: Axial X-ray of the left proximal femur

The fracture was treated with an antegrade femoral nail (Figure 2).

**Figure 3:**
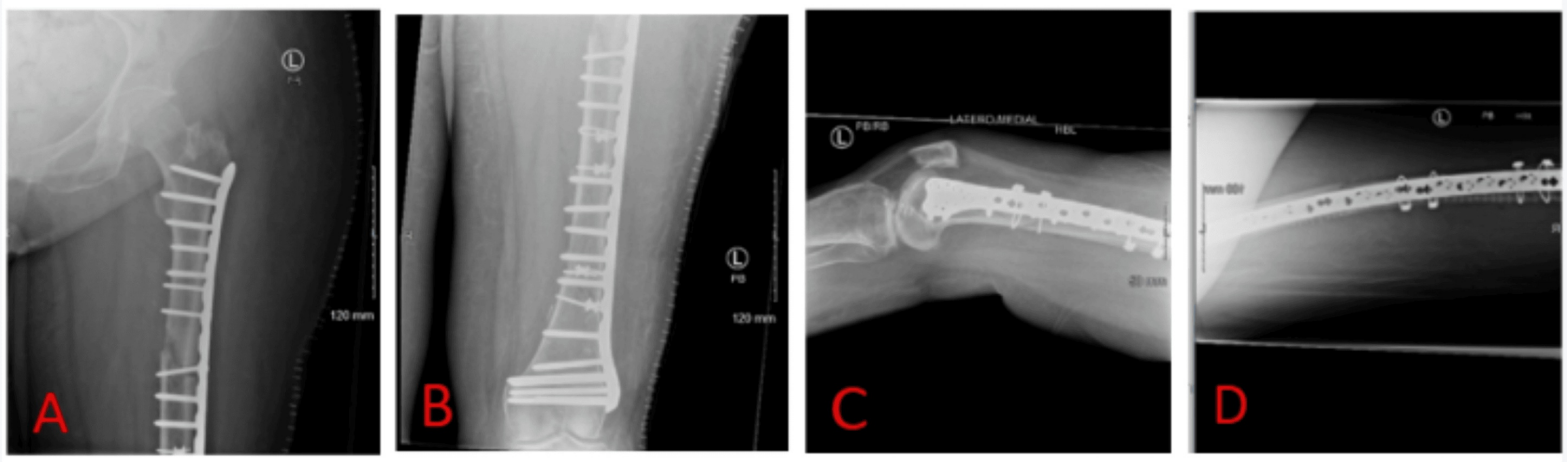
Postoperative X-rays after the first revision surgery (ORIF) from the secondary treating surgeon A: Anteroposterior X-ray of the left proximal femur; B: Anteroposterior X-ray of the left distal femur; C: Lateral X-ray of the left distal femur; D: Lateral X-ray of the left proximal femur ORIF: open reduction and internal fixation

One year and one month after the initial surgery, the patient underwent revision surgery to address the clinically and radiologically confirmed atrophic non-union. An iatrogenic fracture of the distal femur occurred during nail removal, requiring multiple cerclages and a whole-femur-spanning locking plate (Figure 3).

**Figure 4:**
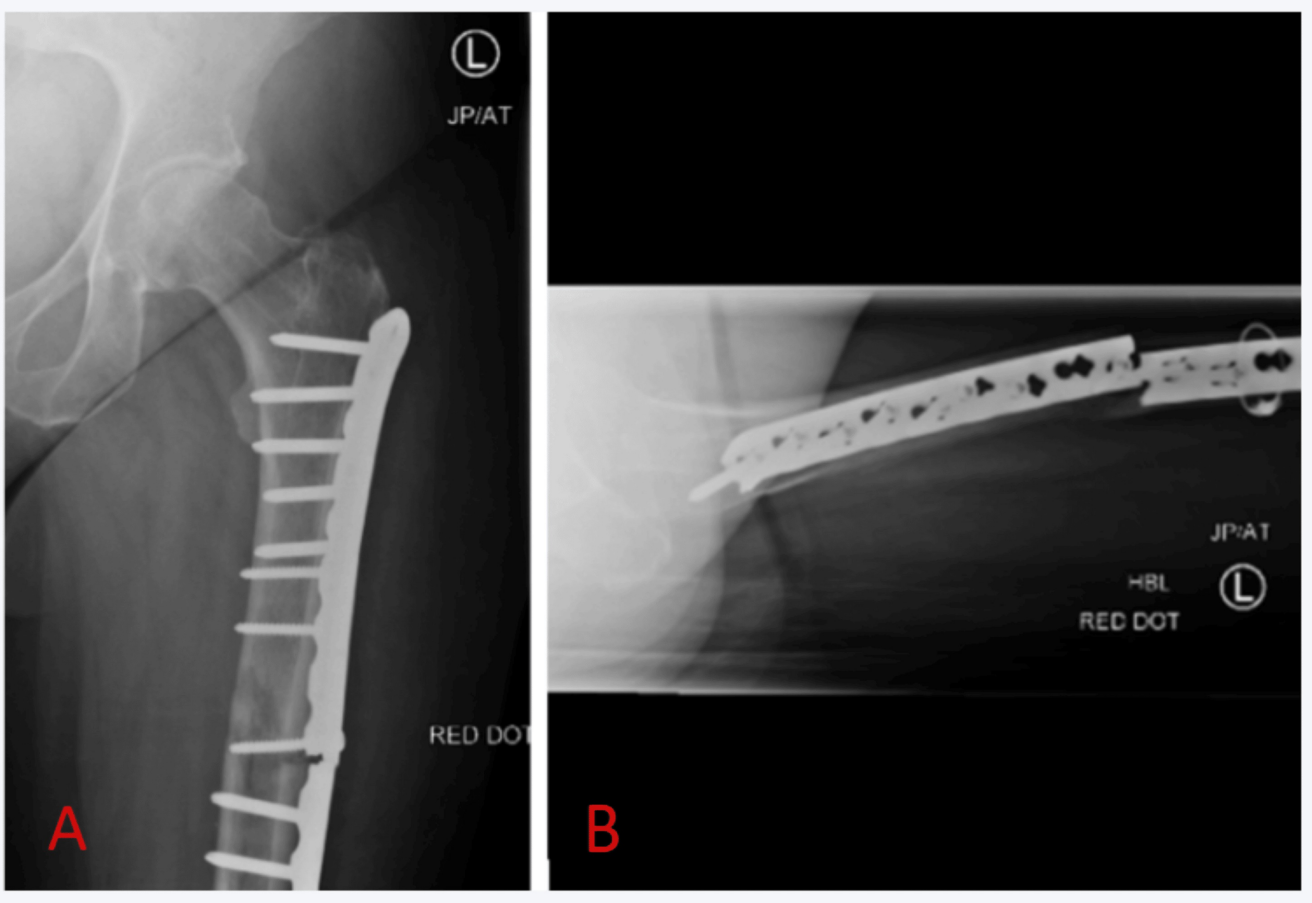
X-rays six weeks after the first revision surgery show the plate breakage at the level of the previous non-union. A: Anteroposterior X-ray of the left proximal femur; B: Axial X-ray of the left proximal femur

Seven weeks later, the plate failed at the site of the previous non-union (Figure 4), and the patient was transferred from a secondary care hospital to our tertiary care centre (a university hospital with a specialized limb reconstruction and bone infection unit). The distal iatrogenic fracture remained stable.

Upon the patient's arrival at our hospital, the physical examination revealed tenderness over the femoral shaft, accompanied by a pronounced visible deformity and slight limb shortening of two to three centimetres with intact neurovascular status. No movement of the knee or hip was possible due to pain. Furthermore, a left-sided dry and clean thigh wound with a dressing in the central region due to occasional ooze was discovered. No further clear clinical signs of infection were noted.

Her comorbidities include chronic obstructive pulmonary disease (COPD), depression, osteopenia, and rheumatoid arthritis. She was taking calcium carbonate/cholecalciferol, famotidine, gabapentin, methotrexate, sertraline, and etoricoxib.

**Figure 5:**
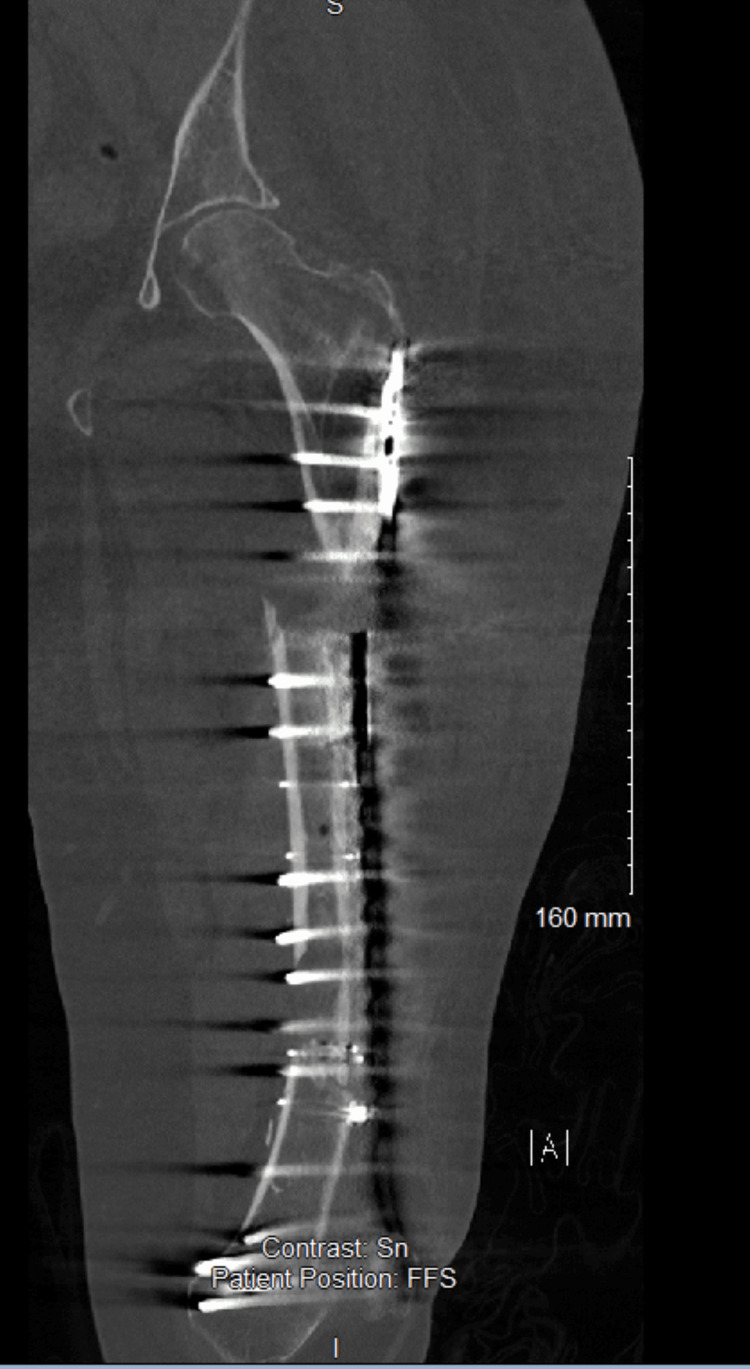
Coronal view of the computed tomography scan of the left femur on arrival at our hospital

The computed tomography scans revealed an atrophic non-union of the proximal femoral shaft with a broken plate and the distal iatrogenic fracture that was healing (Figure 5).

Preoperative blood tests showed elevated levels of C-reactive protein (CRP) at 29 mg/L (reference range: 0-9 mg/L) and an erythrocyte sedimentation rate (ESR) of 109 mm (reference range: 1-19 mm). The white blood cell count (WBC) was normal at 7.9 × 10⁹/L (reference range: 3.9-10.2 × 10⁹/L).

**Figure 6:**
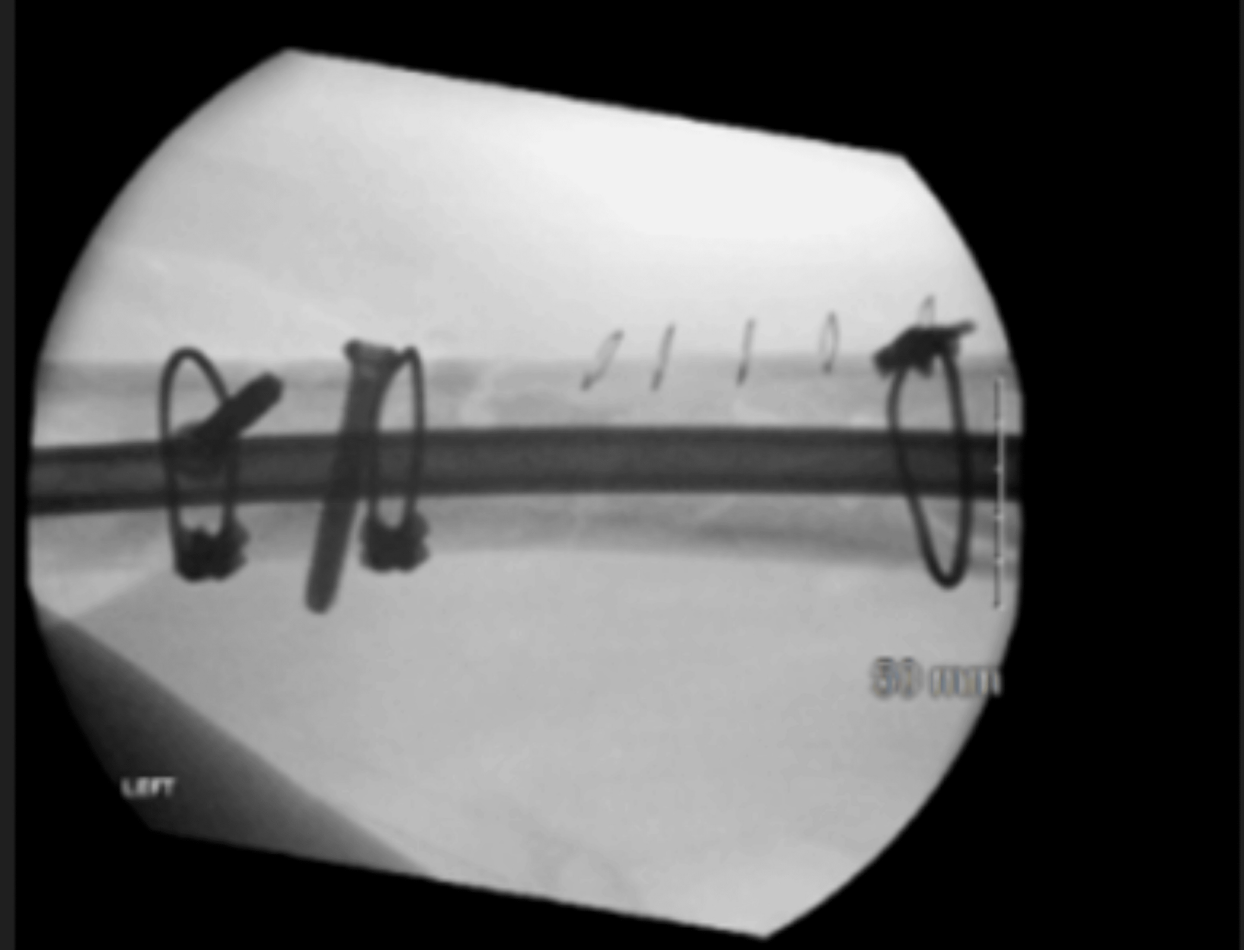
Intraoperative fluoroscopic imaging at the end of our revision surgery after the oblique osteotomy lines have been created and the poller screws and the retrograde femoral nail were inserted.

After obtaining informed consent from the patient, the surgical procedure was performed as follows: Under general anaesthesia, the patient was positioned supine on a radiolucent operating table. An image intensifier was used throughout the procedure. The previous hardware, apart from the cerclages, which were retained, was removed through the existing incisions. The distal femoral fracture was stable. The non-union was resected by creating oblique osteotomy lines using a drill bit and an osteotome. Microbiological samples (five ballotini samples and two sterile container samples) were taken using a non-touch technique [7]. Four poller screws, two in each fragment, were inserted in two orthogonal planes. The canal was reamed to 10.5 mm, and a retrograde femoral nail was inserted (Figure 6). Due to the second revision surgery, antibiotic-impregnated beads (1 g vancomycin and 240 mg gentamicin in 5 ml Stimulan®) were introduced locally.

The initial intraoperatively collected samples (two plain universal containers and five ballotini samples) showed no growth. Twelve days after the second revision surgery, the patient showed signs of a deep wound infection, requiring four additional surgical radical soft tissue debridements while retaining the metalware (on days 12, 23, 30, and 37 following our initial revision surgery). These procedures were accompanied by the simultaneous application of modified irrigated negative pressure with continuous irrigation (MNPCI) wound therapy, followed by meticulous wound closure after each dressing change [8,9]. The negative pressure dressing was removed at the last theatre visit, and the wound was closed over a redivac drain size 10 Fr (French gauge unit) for 72 hours.

The samples obtained during the debridement and application of MNPCI for the deep wound infection revealed vancomycin-resistant enterococcus (VRE) in four out of five ballotini bottles, which were sensitive to daptomycin. The results from these microbiological samples were discussed with the bone infection specialists from the infectious diseases department, and an appropriate antibiotic regimen was established.

Pain management was started immediately after the surgery. Physiotherapy focussing on a range of motion and strengthening exercises, including continuous passive motion (CPM), was initiated on the first postoperative day. Starting on the third postoperative day, the patient mobilized with unrestricted weight-bearing. According to our hospital pathway, prophylaxis of venous thromboembolism (VTE) was administered.

Serial radiographs were obtained postoperatively and every six weeks to monitor healing.

At 11 months, radiographs confirmed union with excellent alignment. The patient regained full weight-bearing ability, with no residual pain or functional limitations at the fracture sites but with significant pain and restrictions due to knee osteoarthritis. Given the VRE-positive samples, the decision was to postpone total knee replacement for at least a few years.

**Figure 7:**
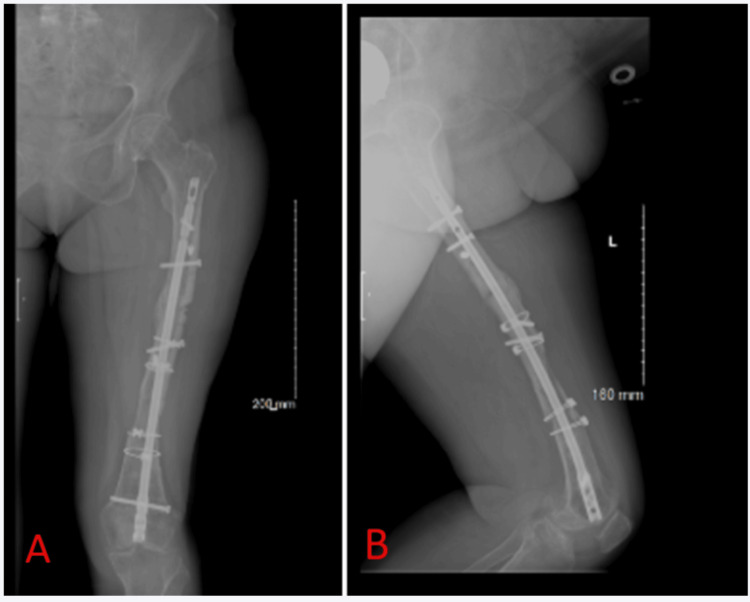
X-rays obtained 19 months postoperatively after our revision surgery A: Anteroposterior X-ray of the left femur; B: Lateral X-ray of the left femur

When this case report was written, the patient was 19 months after the second revision surgery and did not exhibit local or systemic signs of infection, with the non-union and the iatrogenic fracture remaining healed (Figure 7).

## Discussion

Revision surgery with oblique osteotomy and biplanar poller screws enhances mechanical stability and bone healing by converting shearing forces into compression at the non-union site. Acting as blocking screws, poller screws improve intramedullary nailing stability, guide nail placement, prevent malalignment, optimize load distribution, reduce micromotion, and promote controlled compression for better fracture healing [6,10]. By integrating poller screws into our fixation strategy, we aimed to improve construct rigidity and provide a biomechanically stable environment conducive to bone healing. This approach aligns with the principles outlined by Zhou et al., reinforcing the evolving role of poller screws in complex femoral reconstructions [10]. Song [11] demonstrated the positive effect of poller screws combined with an antegrade intramedullary nail in femoral shaft fractures below the isthmus, showing that inserting two additional poller screws after nailing took, on average, 21 minutes of extra time but would significantly reduce the risk of non-union. Similarly, Tennyson et al. [12] confirmed that intramedullary nailing with poller screws results in lower rates of non-union and coronal malalignment compared to nailing alone. Furthermore, Elliot et al. [13] suggest that most non-unions will heal if the correct mechanical environment is established through surgery, without the need for biological adjuncts such as autologous bone grafts. Even though oblique osteotomy does not look advantageous when compared to horizontal osteotomy, we observed that oblique fracture fixations augmented with poller screws have a higher tendency to heal compared to horizontal fractures treated with nails only, regardless of the location of the fracture.

Femoral non-union is often attributed to inadequate stability, biological deficits, or both. The literature review shows a whole range of different concepts, including a one-step procedure using a custom-made intramedullary antibiotic cement-coated carbon nail, as described by Bonicoli et al. [14]; the diamond concept [3]; one- and two-stage procedures [3]; Masquelet techniques [15]; external skeletal fixation with staged bone grafting [16]; and dual implant application [17].

Though the opinions of individual authors vary and are sometimes controversial, it is clear that the complexity of this topic has prevented the establishment of a comprehensive, standardized treatment concept for non-unions. It is also important to note that the success of the aforementioned methods depends on multiple external and patient-related factors, making it difficult to establish a universal definition of success or a standardized treatment recommendation.

From our experience, non-union primarily arises from insufficient mechanical stability, a conclusion supported by McMillan et al. (2017). Their study discusses strategies to prevent delayed healing and non-union in diaphyseal fractures treated with intramedullary nailing, focussing on the importance of accurate fracture reduction, adequate stability, and maintaining intact vascularity for successful bone healing [18].

In the context of the detailed case of femoral non-union managed with an oblique osteotomy, poller screws are particularly advantageous. While facilitating bone realignment and promoting biological healing, the oblique osteotomy may introduce shear forces that could compromise fixation stability. The strategic placement of poller screws in orthogonal planes, as performed in our case, counteracts these shear forces, enhances axial and rotational stability, and prevents excessive micromotion at the osteotomy site. Furthermore, their use optimises the load distribution along the intramedullary nail, reducing the risk of implant failure [6,10,11,12].

We do not regard allografts or autografts as suitable treatment options for infected non-unions of the lower limb, as bone grafting in such cases carries a risk of infection spreading, particularly in non-vascularised grafts. If performed before the infection is fully eradicated, grafting may allow bacterial proliferation, thereby compromising healing. Previous studies [19,20] highlight concerns that bone grafts in infected non-unions could elevate infection risk, potentially hindering recovery and surgical success.

The cerclages were left in situ, as we consider that the advantages of preserving vascularity and soft tissue integrity while minimizing the risk of instability outweigh the benefits of removal, even in the presence of infected metalwork. This approach is supported by studies such as those by Norris et al. [8] and Summers et al. [9].

We believe that mechanical stability, combined with maximizing the bone contact surface of adequately debrided fracture ends through oblique osteotomy, radical infection management with surgical debridement to reduce bacterial load, the use of antibiotic-impregnated beads, multi-sequenced MNPCI wound therapy [8,9] to create a well-vascularized environment, and targeted antibiotic therapy are the key pillars in enabling even an infected fracture to heal and achieve union.

## Conclusions

Our findings underscore the critical role of mechanical stability in the successful management of femoral non-union. Oblique osteotomy combined with biplanar poller screws provided a stable construct after the resection of the atrophic non-union, facilitating bone healing within a reasonable time frame. By transforming shear forces into compression forces, the poller screw technique enhances both biomechanical stability and the biological potential for healing. Furthermore, they improve fixation by guiding intramedullary nail placement, reducing micromotion, and optimising load distribution. While multiple strategies exist for managing femoral non-unions, a standardised treatment concept remains elusive due to patient-specific and external factors. The avoidance of bone grafting in infected femoral non-unions mitigates the risk of bacterial persistence and surgical failure, emphasizing the importance of radical infection control and test-appropriate antibiotics. Ultimately, achieving union in complex infected femoral non-unions requires a multidisciplinary approach that integrates sound mechanical principles with meticulous surgical technique.
